# Do Differences Exist in Impact Test Domains between Youth Athletes with and without an Anterior Cruciate Ligament Injury?

**DOI:** 10.3390/healthcare11202764

**Published:** 2023-10-19

**Authors:** Ashley E. Gureck, Zack Crockett, Brandon W. Barsky, Shenae Samuels, Jeremy S. Frank, Stephen K. Storer, Matthew L. Fazekas

**Affiliations:** 1 Department of Physical Medicine & Rehabilitation, Spaulding Rehabilitation Hospital, Charlestown, MA 02129, USA; 2 Department of Sports Medicine, Kettering Health, Kettering, OH 45429, USA; 3 Memorial Healthcare System, Office of Human Research, Hollywood, FL 33021, USA; 4 Department of Orthopaedic Surgery and Sports Medicine, JoeDiMaggio Children’s Hospital, Hollywood, FL 33021, USA

**Keywords:** ACL, ImPACT, pediatric sports medicine, reaction time

## Abstract

Poor baseline reaction time, as measured via the Immediate Post-Concussion Assessment and Cognitive Test (ImPACT), has been associated with anterior cruciate ligament (ACL) injury risk in adult athletes. Our study sought to determine whether the reaction time and impulse control ImPACT test domains differed between ACL injured and uninjured pediatric athletes. A total of 140 high-school aged athletes comprising 70 athletes who went on to sustain an ACL injury between 2012 and 2018 and 70 age- and sex-matched uninjured controls were included in the study. Mean reaction times were similar for the injured (0.67 s) and uninjured (0.66 s) athletes (*p* = 0.432), and the impulse control scores were also similar for those with (5.67) and without (6.07) an ACL injury (*p* = 0.611). Therefore, neurocognitive risk factors for sustaining an ACL injury in adults cannot necessarily be extrapolated to adolescent athletes. Further research is needed to understand why differences exist between injury risk in youth and adult athletes.

## 1. Introduction

Participation in high school sports continues to rise over the years, setting new records annually. In 2021–2022, participation in high school sports saw only a four percent decline from pre-pandemic highs [1]. According to the National Federation of State High School Associations, roughly 7.6 million athletes participated in high school sports in the 2021–2022 academic year [1]. This number can be compared with about 520,000 NCAA athletes participating in the 2021–2022 sport season [2]. This high participation rate lends itself to a number of musculoskeletal injuries sustained by high school athletes each year, with the anterior cruciate ligament (ACL) injury being one of the most common season, or even career-ending, injuries in this population [3]. When compared to most collegiate athletes, many high school athletes opt to participate in multiple sports. As such, these younger athletes are exposed to a wider array of opportunities for injury, with sports such as soccer, basketball, lacrosse, and football presenting some of the greatest risks for ACL injury [4].

The anterior cruciate ligament, referred to commonly as the ACL, is a band of intra-articular connective tissue that is located within the knee joint. Composed primarily of collagen fibers, the ACL originates from the medial aspect of the lateral femoral condyle and inserts diagonally on the center of the tibial plateau, adjacent to the anterior horn of the lateral meniscus [5]. In addition to providing an anatomic connection between the tibia and femur, the ACL functions to prevent excessive anterior tibial translation, maintain the rotational stability of the tibia, and distribute forces across the intraarticular components [6]. The ACL is able to provide variable tension based on the degree of knee flexion, and thus, is a key contributor to the dynamic stability of the knee [6]. As such, the ACL is a susceptible primary structure to many sport-related injuries.

ACL injuries can be caused by a number of different mechanisms. An injury to the ACL is first classified as either contact or non-contact in nature. As the name implies, contact-related ACL injuries are secondary, where some external force is being applied to the ligament. In comparison, non-contact ACL injuries are caused by non-contact forces such as rotational (plant and pivot) and landing forces [7]. It is believed that as great as 70% of ACL injuries are non-contact in nature [7]. Risk for non-contact ACL injury is mediated by a variety of factors including sex, genetics, anatomy, hormonal regulation, environment, and neurocognition. For example, it is commonly recognized that female athletes are more likely to suffer a non-contact ACL injury when compared to their male counterparts [8,9]. This predisposition to ACL injury is suspected to be secondary to neuromuscular imbalances leading to excessive loads on female athletes’ ligaments [5].

Provided the ACL’s poor innate healing mechanisms and its essential role in knee function, it is not uncommon for athletes, including youth athletes, to undergo reconstructive surgery following ACL rupture. Reconstruction offers athletes greater mechanical stability and limits joint laxity that would otherwise predispose them to recurrent injury. However, it has been demonstrated that young athletes that suffer an ACL injury are nonetheless at a significantly elevated risk of suffering a recurrent injury later in their athletic career. These young athletes have been found to have as high as a 1 in 4 risk of suffering a future ACL injury down the line [10]. Moreover, following ACL reconstruction, young athletes are at a 30 to 40 times greater risk of repeat ACL injury compared with uninjured adolescents [10], most often by way of repeat noncontact injury to the graft [11]. In addition to the risk posed to the injured ligament, athletes suffering an ACL injury are at an increased risk for other joint related pathologies. For example, it is estimated that approximately 20% to 50% of athletes will have evidence of osteoarthritis, most notably at the knee joint, within 10 to 20 years [12]. Additionally, following reconstructive surgery, it is not uncommon for athletes to complain of ongoing pain as a result of direct intraarticular trauma as well as a diminished range of motion and the inability to rest the knee in a natural state of slight hyperextension [12]. Finally, in addition to the significant physical barrier an ACL injury can impose, there is a substantial financial burden as well. It was recently found that the average cost of ACL reconstruction in the United States in the timeframe spanning from July to August 2020 was $29,590 [13]. Given these aforementioned considerations, it is not surprising that these athletes may often have reportedly lower scores on the quality-of-life measurements [12]. As such, the identification of modifiable risk factors for youth ACL injury is essential in order to identify and maintain ACL-protective strategies and inform post-injury rehabilitation protocols.

While some risk factors for ACL injury are inherent, the identification of modifiable domains contributing to ACL injury is an important step in the development of injury prevention and rehabilitation protocols. One modifiable aspect that has received a great deal of attention in the sports injury literature is neurocognition. There is evidence to suggest that certain cognitive skills and central nervous system (CNS) functions may be linked to an increased risk of sports-related injury and subsequent post-injury sports performance in athletes [14]. The baseline neurocognitive performance has important implications on kinetic and kinematic patterns which are believed to increase the risk of ACL injury [15]. Given the fast-paced and unpredictable nature of sport, any deficit in attention, processing speed, or reaction time to correct for errors in coordination impairs the athlete’s ability to maintain neuromuscular control, and therefore, may increase the risk for ACL injury [16,17].

While preparing for athletic participation, it is advised that athletes undergo formal neurocognitive testing in the event of possible brain injury to guide return-to-play. The Immediate Post-Concussion Assessment and Cognitive Test (ImPACT) is a neurocognitive test battery that is designed to aid in the clinical diagnosis of sport-related concussion via the use of five composite scores: verbal memory, visual memory, reaction time, processing speed, and impulse control [18]. The ImPACT test is an approximately 20 min computerized testing battery that is administrated to athletes by qualified providers with an understanding in neurocognitive testing batteries at the beginning of each sport season. Some example tasks include symbol and color matching, pattern and visual recognition, and attention to the numerical data. The test is taken independently and scored directly within the ImPACT software (version 4, https://impacttest.com/), with composite scores in each domain a function of the percent of correctly answered questions. The baseline data are typically then compared to the post-injury test scores at regular intervals to help clinicians evaluate an athlete’s cognitive function and recovery following head injury. Its widespread adoption by schools and sports programs has allowed for effective baseline neurocognitive data collection with minimal added effort or cost to clinicians [18].

Individual composite scores of the ImPACT test including verbal memory, visual memory, visual motor speed, and reaction time have been associated with the risk of lower extremity injury, such as noncontact ACL injury, in college athletes [19,20]. A component of the ImPACT test that does not make up one of the core composites, but rather helps to establish test validity, is impulse control. The impulse control domain is a marker for the number of errors committed throughout the different test phases. Since the impulse control domain of the ImPACT test is traditionally viewed as a marker of test validity, or a “safeguard” to identify athletes who are not putting fourth maximum attention to their ImPACT assessments [21], previous studies have not evaluated its relationship with injury risk. Prior investigations have demonstrated a possible correlation between impulse control disorders and attention deficit disorders [22]. Given the correlation between increased attentional demands and the risk of ACL injury [23,24], poorer impulse control may be associated with an increased incidence of ACL injury. Additionally, no study to our knowledge has examined the relationship between neurocognitive domains and noncontact ACL injury in adolescent athletes.

The present study seeks to compare the relationship between the baseline ImPACT test data and noncontact ACL injury among high school athletes. Given the aforementioned link between certain cognitive deficiencies and sports-related injury, we predict that differences in the reaction time and impulse control domains of the ImPACT test will exist between injured and non-injured high school athletes.

## 2. Methods

### 2.1. Study Design

A case-control design was used to compare the ImPACT neurocognitive test domains and ACL injury in high-school aged athletes within the defined study period, 2012–2018.

A total of 70 student athletes who sustained an ACL injury and underwent surgical intervention for the ACL injury during the study period were first identified as study participants and served as cases for our study. A total of 70 additional student athletes with no known history of ACL injury nor surgical intervention for ACL injury were then matched via age, sex, school, and sport using a 1:1 ratio and subsequently categorized as controls. If multiple uninjured athletes were identified as potential controls, the control was selected randomly.

The dependent variables, reaction time, and impulse control were obtained via the standard yearly pre-season baseline ImPACT tests performed as a pre-requisite for student athlete sport participation. The ImPACT data was extracted with permission from the ImPACT Applications, Inc. website.

The study data were managed using REDCap electronic data capture tools hosted at Memorial Healthcare System (MHS)/Joe DiMaggio Children’s Hospital. The Memorial Healthcare System Institutional Review Board (IRB) determined this study to be exempt from IRB oversight (MHS IRB Project#: MHS.2020.024). Informed consent was waived in this study. A waiver of HIPAA Authorization for Research was approved by MHS IRB.

### 2.2. Subjects

One-hundred and forty student athletes (70 cases plus 70 controls) from various high schools within a single county participated in this study. Athletes participated in a variety of sports and were less than 18 years of age at the time of the pre-injury ImPACT testing.

The clinical and demographic data were extracted from electronic medical records. Potential cases were identified as status-post ACL repair performed by authors SKS and JSF during the years 2012–2018. Athletes were then matched to their corresponding pre-injury ImPACT test scores. All identifying information was kept confidential in a secure database, and the cases and controls were subsequently de-identified for the remainder of the analyses. If an athlete did not have pre-injury ImPACT test data on file, or was over the age of 18 at the time of the pre-injury ImPACT testing, he or she was excluded from the study.

A total of 70 high school student athletes were identified as cases who were less than 18 years of age, underwent ACL repair within the study period, and had pre-injury baseline ImPACT test data on file. If an athlete had multiple ImPACT tests on file, the test data most recent to the ACL injury was used in the analysis. A total of 70 uninjured student athletes with ImPACT test data on file were matched with injured cases on a 1:1 ratio as previously described.

### 2.3. Statistical Analysis

An a priori power analysis was performed using G*Power 3.1.9.2. Power analysis was conducted under the assumptions of a two-tailed test with an alpha error probability of 0.05, a power of 0.80, a medium effect size (Cohen’s d = 0.5), as well as equally sized sample groups. The recommended total sample size for our study, based on the a priori power analysis, was 128 records.

Categorical variables were analyzed using Pearson’s Chi-Square with the results presented as frequencies and proportions. Continuous variables were analyzed using Welch’s t-test with the results presented as means and standard deviations (SD). Summary statistics, such as median, ranges, and interquartile ranges, were also examined and are presented. Effect size, Cohen’s d, was further assessed for the ImPACT composites under study. All analyses were conducted using Stata/SE 15.1. Statistical significance was set at *p* < 0.05 (two-sided).

## 3. Results

### 3.1. Demographics

There were a total of 140 student athletes in this study with a median age of 16 years old for the cases and controls. Half of the student athletes (n = 70) sustained an ACL injury and underwent surgical repair while the other 70 athletes had no known history of ACL injury. A total of 38 cases of ACL injury and repair (54.3%) were female, and 32 cases (45.7%) were male. Table 1 presents the baseline characteristics and shows the similarity in the distribution of demographics by injury status as a result of the study’s age- and sex-control matching.

### 3.2. Outcomes

Table 2 summarizes the differences observed in the reaction time and impulse control domains in the injured versus matched uninjured study participants. Reaction times in the injured group ranged from 0.48 to 1.26 s and 0.50 to 1.12 s in the uninjured group. Impulse control scores ranged from 0 to 38 in the injured group and 0 to 20 in the uninjured group. No statistically significant differences were observed between the ACL-injured and uninjured groups.

**Figure 1:**
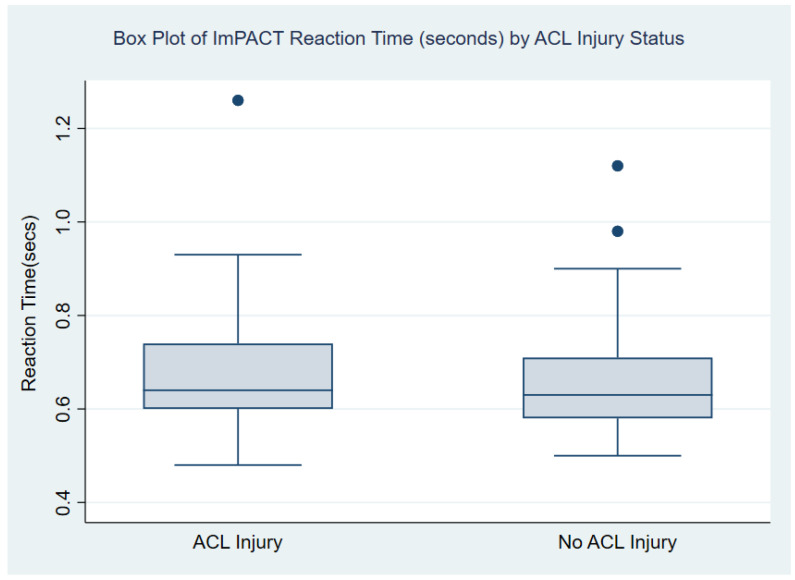
Box slot of summary statistics for ImPACT reaction time by ACL injury status. Boxes include the first quartile (Q1) to the third quartile (Q3) with a horizontal line in the middle of the box to denote the median. In each group, the upper and lower whiskers of the box plot are the largest and smallest reaction times, respectively, that are within 1.5 times the IQR. Filled circles were used to denote outliers on the figure (i.e., data points below Q1 − 1.5*IQR or above Q3  +  1.5*IQR).

**Figure 2:**
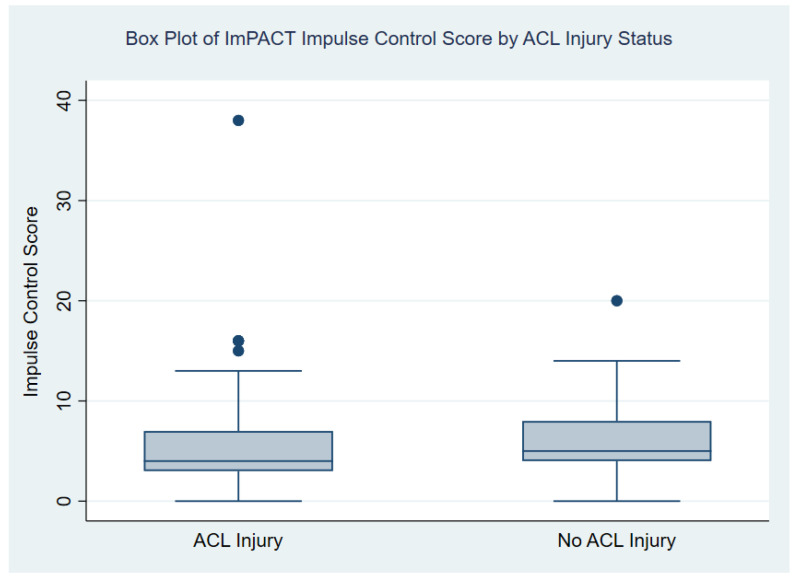
Box plot of summary statistics for ImPACT impulse control by ACL injury status. Boxes include the first quartile (Q1) to the third quartile (Q3) with a horizontal line in the middle of the box to denote the median. In each group, the upper and lower whiskers of the box plot are the largest and smallest impulse control scores, respectively, that are within 1.5 times the IQR. Filled circles were used to denote outliers on the figure (i.e., data points below Q1 − 1.5*IQR or above Q3  +  1.5*IQR).

In the analysis of the ImPACT composite of reaction time, the results showed no statistically significant difference between the mean reaction times of the injured and uninjured student athletes. Mean reaction times were very similar among the injured (0.67 s) and uninjured (0.66 s) student athletes (*p* = 0.432). While the mean impulse control scores were slightly higher for the uninjured athletes (6.07) compared to injured athletes (5.67), this difference was not statistically significant (*p* = 0.611). The median and interquartile ranges (IQR) of the reaction time and impulse control domains by injury status are also presented in Table 2, as well as in Figure 1 and Figure 2.

In examining the effect size, the results further suggest that the observed differences for the reaction time (Cohen’s d = 0.13) and impulse control score (Cohen’s d = 0.09) between the two groups were negligible.

## 4. Discussion

### 4.1. Conclusions

Our findings suggest that, unlike in the adult population, the reaction time measured via the ImPACT test does not differ among adolescent athletes who previously sustained an ACL injury and those who had not. Our study’s results yielded a small effect size, Cohen’s d = 0.13, underscoring the marginal difference we observed between the two groups. Additionally, while attention has been demonstrated to play a role in the risk for ACL injury in the adult population, the difference we observed in the impulse control scores between injured adolescent athletes compared to the healthy controls was trivial with a small effect size, Cohen’s d = 0.09. An ACL tear is undoubtedly a devastating injury for athletes of all experience levels. The identification of modifiable risk factors for ACL injury in the high school athlete population is an important step toward providing targeted intervention toward injury prevention in high-risk athletes and informing rehabilitation protocols after an injury is sustained. While existing studies have demonstrated relationships in ImPACT test domains with the risk of noncontact ACL injury in the adult athlete [19,20], this is the first study to date that has examined this relationship among adolescent athletes.

### 4.2. Alternative Factors Contributing to Injury in Youth Athletes

There are multitude of factors that ultimately contribute to an athlete’s sports performance and injury risk. Two such factors, attention and focus, both contribute to athletic success and can be implicated in sport-related injury. Specifically, an athlete’s ability to maintain what is referred to as external focus is largely related to athletic performance. External focus is one’s ability to channel attention to external cues in the environment throughout an activity [25]. It has been shown that sprinters that were able to channel their focus externally prior to competing in an event were able to improve their sprint times [26]. Similarly, gymnasts were able to produce superior movement patterns and jump-heights when they were placed in conditions encouraging external focus [27]. It is believed that external focus may help athletes promote an automatization of movement patterns and limit interference or possible constraints from less-automatic cognitive processing [28]. Importantly, external focus has been indicated as an important factor in sports involving change-of-direction. Athletes competing in change-of-direction sports and sports with acceleration tasks that involve diagonal cutting may improve their performance by shifting from an internal or neutral focus to a more externally focused pattern [29]. This is significant as the mechanism of ACL injury often incorporates cutting or change-of-direction style movements [29].

Interestingly, while external focus has been demonstrated to improve performance in adult athletes, there may be evidence that an external center of foci may counterintuitively worsen athletic performance in adolescent athletes. Investigators found that novice adolescent athletes showed a worsened performance, as measured using muscle activation and movement latency, when attempting to kick a soccer ball at a given target [30]. The authors go on to hypothesize that younger, more novice athletes are not yet proficient in their sport-specific motor control. In the setting of diminished motor coordination, an athlete may require a shift toward more internal focus so as to achieve proper muscular control in a given movement. Improper muscle activation and coordination may predispose these younger athletes to a greater risk of sports-related injury, notably non-contact ACL injury [31]. This discussion on external focus and its relationship to injury risk relates to our study population as those scoring lower on ImPACT impulsivity testing may have a diminished capacity for external focus.

As previously stated, there may exist a link between impulse control disorders and attention deficit disorders [22]. Further sports-specific correlations have been made linking reduced impulse control and poor motor control. Following a sports-related concussion, adolescent athletes were shown to have worsened Perception-Action Coupling Task (PACT) and Barret Impulsivity Scale (BIS) as well as attention and cognitive instability scores [32]. The PACT score here serves as a measure of motor control, alertness, and reaction time. This indicates a potential relationship between impulsivity and motor control. Similarly, in an investigation of NCAA Division-I athletes, 50.4% with a known history of Attention Deficit Hyperactivity Disorder (ADHD) reported a history of at least one prior concussion compared to 14.4% of athletes without a known history of ADHD [33]. This may offer evidence to the hypothesis that diminished attention and impulsivity predispose athletes to sports-specific injuries. These investigations are, however, only correlative in nature. It is clear based on our results that further investigations may be needed to clarify the role that impulsivity and attention have on motor functioning.

Aside from attention and focus, there are a number of factors that can ultimately predispose an athlete to injury. Individual nuances, such as age, sex, race, and experience level, need to be accounted for when establishing the risk for specific sports injuries. There is evidence that frequently neglected factors, such as shoe–surface interactions, hormonal variations, and specific weather patterns, may all be implicated as contributory factors to ACL injuries [34]. For example, it has been demonstrated that white athletes are more likely to suffer an ACL injury when compared to black athletes [35]. Similarly, evidence suggests that age-related differences such as diminished joint position sense and muscle response time may affect the injury rate and mechanism. These neuromuscular differences may very well translate to differences in injuries that are sustained by athletes of various ages. When comparing youth and adult athletes, there were found to be differences in the type and mechanism of injury sustained while undergoing resistance training [36]. Therefore, individualistic and population differences that exist between adolescent and adult athletes may contribute to the differences seen in ImPACT scoring and injury risk.

Similarly, another important aspect to consider when interpreting this data is the complex nature of sports-related injuries. As previously discussed, musculoskeletal injuries are often multifactorial in nature and their etiology can stem from a number of independent factors. It has been shown that designing screening tests to assess for individual variances that predispose athletes to injuries can be quite difficult [37]. Not only must a survey capture the various elements that may predict injury, but must also account for differences in respondents’ background characteristics. In previous studies, the Functional Movement Screening (FMS) test, which has been shown to correlate with injury risk in adults, was not helpful in predicting future injuries in younger athletes [38,39]. ImPACT testing works to address these possible discrepancies by offering both standard and pediatric-specific ImPACT tests. Despite this effort, ImPACT testing measures may potentially prove useful in predicting injury in the adult population while still lacking validity in the pediatric population, as demonstrated by our results.

Reaction time and attention have previously been demonstrated to correlate with ACL injury risk in the adult population. While there is evidence that there may be a link between the visual–spatial attention performance and lower extremity musculoskeletal injury risk in adolescent athletes, this was not able to be confirmed in our investigation of adolescent athletes [40]. However, one recent scoping review identified larger factors such as the training load, playing in competition, and prior injury as notable risk factors for sustaining injury in adolescent athletes competing at the non-elite level [41]. Some investigators go on to propose that sports-related injuries include an even greater multitude of variables. Proposed variables include sleep, nutrition, training load, training intensity, prior sport exposure, and competence-based self-esteem [42]. Regardless of the exact factors that go into sports-related injuries, they are injuries that are generally seen as multifactorial in nature, often making prediction-based models difficult. It is evident that there exists a significant need for future investigations to help further clarify the role that variables such as reaction time and impulse control have on adolescent sport injury risk and how these interact with the other biomechanic and psychosocial variables in adolescent athletes.

### 4.3. Limitations

While our results suggest that reaction time and impulse control are not useful indices for identifying youth athletes potentially at risk for ACL injury, our study only allows for speculation as to why this difference exists between adult and adolescent athletes. For example, ACL injury in adolescent athletes may be driven to a greater extent by other aforementioned alternative risk factors when compared with adults. A considerable role may also be played by other neurocognitive domains such as verbal or visual memory, or visual motor speed, which this study did not seek to investigate. Our investigation also did not consider the experience level of the athletes that were included. Given the recent evidence suggesting that external focus may impair motor coordination in adolescent athletes at various experience levels, this may serve as an important confounding variable.

Additionally, our inquiry of the impulse control composite was performed under the assumption that impulse control serves as a reliable measure of attention. The impulse control assessment is a validity measure which involves a series of simple “go/no-go” and color matching tasks that serve to ensure an athlete’s maximal effort and attentiveness to the task at hand. It is well known that children with attention deficit hyperactivity disorder (ADHD) exhibit impulsivity [32], and the recent literature has demonstrated that student athletes with ADHD more frequently fall below the ImPACT validity score thresholds than those without neurocognitive deficits [43,44]. As such, we conducted this study on the basis that the impulse control composite is a reliable surrogate of attention on the ImPACT test. However, because attention has been previously documented as a significant factor in the maintenance of neuromuscular control, and therefore, the avoidance of ACL injury [23,24]. The insignificant difference in impulse control composites we observed between our injured and uninjured groups may call into question the validity of our assumption. This could indicate that attention may in fact contribute to ACL injury risk with the ImPACT impulsivity domain serving as a nonoptimal measure of attention.

Overall, the ImPACT test is considered a reliable, sensitive tool to assist providers in the clinical diagnosis of concussion and inform decisions on return-to-play. Its widespread use, accessibility, and affordability make the ImPACT test a convenient method of data collection as it pertains to concussion and related neurocognitive domains. However, recent studies have questioned the validity and specificity of discriminant domains of the ImPACT test, especially as they compare to traditional neuropsychological test batteries [18,45]. Our study may have benefitted from the use of neurocognitive testing batteries with greater diagnostic precision to identify more subtle differences in reaction time and attention or impulse control in adolescent athletes with and without ACL injury.

Finally, our study included a relatively small sample of local athletes. As such, these results may not necessarily be generalized to all youth athletes. As was previously discussed, individual and population differences may greatly impact an athlete’s biomechanics and injury risk. For this reason, regression modeling with a larger more heterogenous group may better elucidate these differences and determine whether a specific threshold in baseline neurocognitive domains exists that confers an increased incidence of ACL injury. Finally, understanding the predictive value of baseline neurocognitive domains on future injury risk would be more effective using a prospective study design.

### 4.4. Future Directions

Our study provides new information pertaining to how reaction time and attention may impact the ACL injury rate in adolescent athletes. In the context of the existing literature, we provide new data that may suggest that reaction time and attention may not serve as important factors in injury risk when compared to the adult athlete counterparts. Additionally, our results shed light on the need for improved screening tools to identify youth athletes at risk for ACL injury. Further investigation is warranted to identify specific neurocognitive domains that may be useful in identifying adolescent athletes at an increased risk for ACL injury. Studies should seek to identify differences that exist between the adult and adolescent neurocognitive domains that may influence the risk for injury. Validated neuropsychological testing batteries, in addition to the ImPACT test, may be useful in recognizing subtle differences that exist between these populations. Furthermore, prospective studies are needed to evaluate the modifiable risk factors for developing ACL injuries in the pediatric population. It will be important that investigators consider factors such as the sport-specific experience level in secondary analysis. Finally, additional research is needed to inform how these results should influence future injury prevention and rehabilitation programs.

## Figures and Tables

**Table 1:** Baseline Characteristics of Student Athletes Stratified by ACL Injury Status.

	ACL Injury (%)	No ACL Injury (%)	*p*-Value
N	70 (50)	70 (50)	NE
Sex			1.000
Male	32 (45.7)	32 (45.7)	
Female	38 (54.3)	38 (54.3)	
Age (Years)			
Mean ± SD	15.4 ± 1.2	15.6 ± 1.1	0.461
Median (IQR)	16 (14–17)	16 (14–17)	NE

NE = Not Estimated. IQR = Interquartile Range. Results based on Pearson’s chi-squared test for categorical variables, and Welch’s *t*-test for independent samples for continuous variables. No statistically significant difference was found in age or sex of participants. Median values are presented with IQR where the lower bound is the 25th percentile and the upper bound is the 75th percentile.

**Table 2:** ImPACT Reaction Time and Control Scores by ACL Injury Status.

ImPACT Composite	ACL Injury	No ACL Injury	*p*-Value	Cohen’s d
Reaction Time				
Mean ± SD	0.67 ± 0.12	0.66 ± 0.12	0.432	0.13
Median (IQR)	0.64 (0.51–0.87)	0.63 (0.51–0.89)	NE	NE
Impulse Control				
Mean ± SD	5.67 ± 5.38	6.07 ± 3.75	0.611	0.09
Median (IQR)	4 (1–15)	5 (1–12)	NE	NE

NE = Not Estimated. IQR = Interquartile Range. Results based on Welch’s *t*-test for independent samples. No statistically significant difference was found between ACL-injured and ACL-uninjured athletes for either reaction time or impulse control domains. Median values are presented with IQR where the lower bound is the 25th percentile and the upper bound is the 75th percentile.

## Data Availability

The data that support the findings of this study are available by reasonable request from the authors, M.L.F. and S.S., and with the permission of ImPACT Applications, Inc and Nova Southeastern University, who holds the database. Restrictions apply to the availability of these data which contain personal demographic data and PHI for minors under the age of 18.

## References

[1] NFHS (2022). NFHS Releases First High School Sports Participation Survey in Three Years. https://www.nfhs.org/articles/nfhs-releases-first-high-school-sports-participation-survey-in-three-years/.

[2] NCAA Media Center (2022). NCAA Student-Athletes Surpass 520,000, Set New Record. https://www.ncaa.org/news/2022/12/5/media-center-ncaa-student-athletes-surpass-520-000-set-new-record.aspx.

[3] Tirabassi J., Brou L., Khodaee M., Lefort R., Fields S.K., Comstock R.D. (2016). Epidemiology of High School Sports-Related Injuries Resulting in Medical Disqualification: 2005–2006 through 2013–2014 Academic Years. Am. J. Sports Med..

[4] Bram J.T., Magee L.C., Mehta N.N., Patel N.M., Ganley T.J. (2021). Anterior Cruciate Ligament Injury Incidence in Adolescent Athletes: A Systematic Review and Meta-Analysis. Am. J. Sports Med..

[5] Yoo H., Marappa-Ganeshan R. (2023). Anatomy, Bony Pelvis and Lower Limb, Knee Anterior Cruciate Ligament. StatPearls.

[6] Domnick C., Raschke M.J., Herbort M. (2016). Biomechanics of the Anterior Cruciate Ligament: Physiology, Rupture and Reconstruction Techniques. World J. Orthop..

[7] Griffin L.Y., Agel J., Albohm M.J., Arendt E.A., Dick R.W., Garrett W.E., Garrick J.G., Hewett T.E., Huston L., Ireland M.L. (2000). Noncontact Anterior Cruciate Ligament Injuries: Risk Factors and Prevention Strategies. J. Am. Acad. Orthop. Surg..

[8] Stanley L.E., Kerr Z.Y., Dompier T.P., Padua D.A. (2016). Sex Differences in the Incidence of Anterior Cruciate Ligament, Medial Collateral Ligament, and Meniscal Injuries in Collegiate and High School Sports: 2009–2010 Through 2013–2014. Am. J. Sports Med..

[9] Sutton K.M., Bullock J.M. (2013). Anterior Cruciate Ligament Rupture: Differences between Males and Females. J. Am. Acad. Orthop. Surg..

[10] Wiggins A.J., Grandhi R.K., Schneider D.K., Stanfield D., Webster K.E., Myer G.D. (2016). Risk of Secondary Injury in Younger Athletes After Anterior Cruciate Ligament Reconstruction: A Systematic Review and Meta-Analysis. Am. J. Sports Med..

[11] Gans I., Retzky J.S., Jones L.C., Tanaka M.J. (2018). Epidemiology of Recurrent Anterior Cruciate Ligament Injuries in National Collegiate Athletic Association Sports: The Injury Surveillance Program, 2004–2014. Orthop. J. Sports Med..

[12] Sepúlveda F., Sánchez L., Amy E., Micheo W. (2017). Anterior Cruciate Ligament Injury: Return to Play, Function and Long-Term Considerations. Curr. Sports Med. Rep..

[13] Lee J., Guzek R.H., Shah N.S., Lawrence J.T.R., Ganley T.J., Shah A.S. (2022). How Much Will My Child’s ACL Reconstruction Cost? Availability and Variability of Price Estimates for Anterior Cruciate Ligament Reconstruction in the United States. J. Pediatr. Orthop..

[14] Piskin D., Benjaminse A., Dimitrakis P., Gokeler A. (2022). Neurocognitive and Neurophysiological Functions Related to ACL Injury: A Framework for Neurocognitive Approaches in Rehabilitation and Return-to-Sports Tests. Sports Health.

[15] Herman D.C., Barth J.T. (2016). Drop-Jump Landing Varies With Baseline Neurocognition: Implications for Anterior Cruciate Ligament Injury Risk and Prevention. Am. J. Sports Med..

[16] Swanik C.B. (2015). Brains and Sprains: The Brain’s Role in Noncontact Anterior Cruciate Ligament Injuries. J. Athl. Train..

[17] Grooms D.R., Onate J.A. (2016). Neuroscience Application to Noncontact Anterior Cruciate Ligament Injury Prevention. Sports Health.

[18] Alsalaheen B., Stockdale K., Pechumer D., Broglio S.P. (2016). Validity of the Immediate Post Concussion Assessment and Cognitive Testing (ImPACT). Sports Med. Auckl. N. Z..

[19] Swanik C.B., Covassin T., Stearne D.J., Schatz P. (2007). The Relationship between Neurocognitive Function and Noncontact Anterior Cruciate Ligament Injuries. Am. J. Sports Med..

[20] Wilkerson G.B. (2012). Neurocognitive Reaction Time Predicts Lower Extremity Sprains and Strains. Int. J. Athl. Ther. Train..

[21] Iverson G.L., Lovell M.R., Collins M.W. (2003). Interpreting Change on ImPACT Following Sport Concussion. Clin. Neuropsychol..

[22] Specker S.M., Carlson G.A., Christenson G.A., Marcotte M. (1995). Impulse Control Disorders and Attention Deficit Disorder in Pathological Gamblers. Ann. Clin. Psychiatry Off. J. Am. Acad. Clin. Psychiatr..

[23] Almonroeder T.G., Kernozek T., Cobb S., Slavens B., Wang J., Huddleston W. (2019). Divided Attention during Cutting Influences Lower Extremity Mechanics in Female Athletes. Sports Biomech..

[24] Shultz S.J., Schmitz R.J., Benjaminse A., Collins M., Ford K., Kulas A.S. (2015). ACL Research Retreat VII: An Update on Anterior Cruciate Ligament Injury Risk Factor Identification, Screening, and Prevention. J. Athl. Train..

[25] Park S.H., Yi C.W., Shin J.Y., Ryu Y.U. (2015). Effects of External Focus of Attention on Balance: A Short Review. J. Phys. Ther. Sci..

[26] Li D., Zhang L., Yue X., Memmert D., Zhang Y. (2022). Effect of Attentional Focus on Sprint Performance: A Meta-Analysis. Int. J. Environ. Res. Public. Health.

[27] Abdollahipour R., Wulf G., Psotta R., Palomo Nieto M. (2015). Performance of Gymnastics Skill Benefits from an External Focus of Attention. J. Sports Sci..

[28] Neumann D.L. (2019). A Systematic Review of Attentional Focus Strategies in Weightlifting. Front. Sports Act. Living.

[29] McNicholas K., Comyns T.M. (2020). Attentional Focus and the Effect on Change-of-Direction and Acceleration Performance. J. Strength Cond. Res..

[30] Uslu S., Çetin Özdoğan E. (2023). External Focus Reduces Accuracy and Increases Antagonist Muscle Activation in Novice Adolescent Soccer Players. Mot. Control.

[31] Bencke J., Aagaard P., Zebis M.K. (2018). Muscle Activation During ACL Injury Risk Movements in Young Female Athletes: A Narrative Review. Front. Physiol..

[32] Winstanley C.A., Eagle D.M., Robbins T.W. (2006). Behavioral Models of Impulsivity in Relation to ADHD: Translation between Clinical and Preclinical Studies. Clin. Psychol. Rev..

[33] Alosco M.L., Fedor A.F., Gunstad J. (2014). Attention Deficit Hyperactivity Disorder as a Risk Factor for Concussions in NCAA Division-I Athletes. Brain Inj..

[34] Brophy R.H., Silvers H.J., Mandelbaum B.R. (2010). Anterior Cruciate Ligament Injuries: Etiology and Prevention. Sports Med. Arthrosc. Rev..

[35] Devana S.K., Solorzano C., Nwachukwu B., Jones K.J. (2022). Disparities in ACL Reconstruction: The Influence of Gender and Race on Incidence, Treatment, and Outcomes. Curr. Rev. Musculoskelet. Med..

[36] Myer G.D., Quatman C.E., Khoury J., Wall E.J., Hewett T.E. (2009). Youth versus Adult “Weightlifting” Injuries Presenting to United States Emergency Rooms: Accidental versus Nonaccidental Injury Mechanisms. J. Strength Cond. Res..

[37] Schweizer N., Strutzenberger G., Franchi M.V., Farshad M., Scherr J., Spörri J. (2022). Screening Tests for Assessing Athletes at Risk of ACL Injury or Reinjury-A Scoping Review. Int. J. Environ. Res. Public. Health.

[38] Bushman T.T., Grier T.L., Canham-Chervak M., Anderson M.K., North W.J., Jones B.H. (2016). The Functional Movement Screen and Injury Risk: Association and Predictive Value in Active Men. Am. J. Sports Med..

[39] Bring B.V., Chan M., Devine R.C., Collins C.L., Diehl J., Burkam B. (2018). Functional Movement Screening and Injury Rates in High School and Collegiate Runners: A Retrospective Analysis of 3 Prospective Observational Studies. Clin. J. Sport Med. Off. J. Can. Acad. Sport Med..

[40] Avedesian J.M., McPherson A.L., Diekfuss J.A., Barber Foss K.D., Hogg J.A., Zuleger T.M., Dufek J.S., Myer G.D. (2022). Visual-Spatial Attentional Performance Identifies Lower Extremity Injury Risk in Adolescent Athletes. Clin. J. Sport Med. Off. J. Can. Acad. Sport Med..

[41] Sainsbury D., Downs J., Nettoi K. (2023). Leanda McKenna Factors Associated With Sports Injuries in Adolescents Who Play Team Sports at a Nonelite Level: A Scoping Review. J. Orthop. Sports Phys. Ther..

[42] von Rosen P., Frohm A., Kottorp A., Fridén C., Heijne A. (2017). Multiple Factors Explain Injury Risk in Adolescent Elite Athletes: Applying a Biopsychosocial Perspective. Scand. J. Med. Sci. Sports.

[43] Manderino L., Gunstad J. (2018). Collegiate Student Athletes With History of ADHD or Academic Difficulties Are More Likely to Produce an Invalid Protocol on Baseline ImPACT Testing. Clin. J. Sport Med. Off. J. Can. Acad. Sport Med..

[44] Maietta J.E., Barchard K.A., Kuwabara H.C., Donohue B.D., Ross S.R., Kinsora T.F., Allen D.N. (2021). Influence of Special Education, ADHD, Autism, and Learning Disorders on ImPACT Validity Scores in High School Athletes. J. Int. Neuropsychol. Soc. JINS.

[45] Thoma R.J., Cook J.A., McGrew C., King J.H., Pulsipher D.T., Yeo R.A., Monnig M.A., Mayer A., Pommy J., Campbell R.A. (2018). Convergent and Discriminant Validity of the ImPACT with Traditional Neuropsychological Measures. Cogent Psychol..

